# Influences of decision preferences and health literacy on temporomandibular disorder treatment outcome

**DOI:** 10.1186/s12903-022-02420-x

**Published:** 2022-09-05

**Authors:** Jeong-Hyun Kang

**Affiliations:** Clinic of Oral Medicine and Orofacial Pain, Institute of Oral Health Science, Ajou University School of Medicine, 164, Worldcup-ro, Yeongtong-gu, Suwon, Gyeonggi-do 16499 Republic of Korea

**Keywords:** Temporomandibular joint disorders, Health literacy, Decision-making, Pain, Treatment outcome

## Abstract

**Background:**

Shared decision-making is defined as the process by which physicians and informed patients make a shared medical decision, taking into account the preferences and values of the patients. It is well known that shared decision-making practices improve both clinicians’ and patients’ satisfaction and lead to better treatment outcomes**.** The aim of the study was to assess associations between patients’ roles in decision-making, health literacy levels, and treatment outcomes of temporomandibular disorders (TMDs).

**Methods:**

In total, 131 participants were enrolled. Participants underwent interview and physical examination at baseline and six months after TMD management. TMD was diagnosed according to Diagnostic Criteria/TMD criteria. Myofascial trigger points were bilaterally evaluated in the two masticatory muscles including the temporalis and masseter muscles. The roles that participants preferred to play or had perceived during decision-making and their health literacy levels were assessed using Control Preferences Scale and Newest Vital Sign, respectively.

**Results:**

Participants who perceived themselves as occupying active roles in decision-making showed higher health literacy levels than those with passive perceived roles. Participants with appropriate health literacy showed higher perceived participation levels in decision-making than did those with limited health literacy. The extent of subjective symptomatic improvement after six months of treatment showed significant associations with perceived role in decision-making, despite lack of significant relationships between perceived role in decision-making and the extent of improvement of objective parameters.

**Conclusion:**

Active participation of patients in decision-making improves the satisfaction but limited health literacy constitutes barriers to effective patient engagement during TMD management.

**Supplementary Information:**

The online version contains supplementary material available at 10.1186/s12903-022-02420-x.

## Background

Temporomandibular disorder (TMD) is defined as a collection of conditions affecting the temporomandibular joint (TMJ), masticatory muscles, and surrounding structures, and it could manifest with high degree of complexity through a diverse and wide spectrum of etiology [1]. The pathophysiology of TMD is multifactorial, meaning that no one contributing factor or single model can explain its development and progression [1]. Therefore, to properly manage the TMD patients, it is essential to help inform the patients about the properties of the disease and allow the patients to participate actively in the treatment process.

Shared decision-making is defined as the process by which physicians and informed patients make a shared medical decision, taking into account the preferences and values of the patients [2]. It is well known that shared decision-making practices improve both clinicians’ and patients’ satisfaction and lead to better treatment outcomes, fewer decision conflicts, and a better physician–patient relationship [2–5]. The active participation of patients in decision-making is important in limiting the potential for misunderstandings of patients’ preferences [6]. However, sparse studies have attempted to reveal the associations between shared decision-making, treatment outcomes, and the satisfaction on treatment outcomes of dentists and patients as they relate to TMD management.

Health literacy refers to a patient’s ability to gather, understand, and act on health information and make informed health decisions [7]. The relationships between levels of health literacy, shared decision-making, and participation in treatment procedures have been reported [8–14], with several studies demonstrating that patients with limited health literacy asked fewer questions during office visits [10, 13] and that active involvement in decision-making was associated with higher health literacy and educational achievement [9, 12]. In some situations, patients with limited health literacy have barriers to getting proper management, including non-adherence to treatment plans and medical regimens, as well as poor self-care owing to their limited understanding of the status of their health conditions [8, 11]. As a result, patients with lower health literacy might be at risk of a diminished understanding of a medical encounter and lower treatment satisfaction [14]. An interesting point was that even though patients with lower health literacy showed passive participation in decision-making, they were willing to actively participate in the medical decision-making and treatment procedures [12]. Therefore, screening patients’ background levels of health literacy before treatment and providing adequate tools to encourage to increase their medical knowledge would be recommended to facilitate the active participation of patients in decision-making, thereby promoting better treatment outcomes.

The influences of the patients’ participation in shared decision-making on treatment success and satisfaction, as well as the role of health literacy in patients’ participation and shared decision-making have been demonstrated in several conditions [9–14]. However, to the best of the knowledge, of the field, the impacts of shared decision-making and the level of a patient’s health literacy on TMD treatment have not yet been elucidated. Therefore, the aim of the present study was to assess the associations between TMD patients’ roles in decision-making, their degrees of health literacy, their satisfaction with the TMD management, and their treatment outcomes.

## Materials and methods

### Participants

This study is a cross-sectional study. A total of 131 Korean patients (15 males, 116 females; mean age, 37.9 ± 14.7 years; age range 19–76 years) who visited the TMJ·Orofacial Pain Clinic at a tertiary medical center in from July 2019 to July 2020 were enrolled. Patients with a history of head and neck trauma within 12 months of study entry; autoimmune diseases such as systemic lupus erythematosus, rheumatoid arthritis, fibromyalgia, and ankylosing spondylitis; craniofacial anomalies, including cleft lip and palpate; neurodegenerative disorders; history of temporomandibular joint surgery; routine intake of antipsychotic drugs; and communication incapability were excluded. Patients who were associated with worker’s compensation such as private or public insurance were also excluded from the study owing to secondary gain.

All participants were interviewed and examined by a single TMD and orofacial pain specialist (JHK). The participants underwent an interview and a physical examination at baseline (T0) and six months after TMD management (T1). Assessments of both the roles that participants preferred to play, or had perceived themselves as playing during the decision-making process and the levels of health literacy of the participants were performed at T1.

The research protocol was reviewed and found to be in compliance with the Helsinki Declaration and was approved by the Institutional Review Board of the Ajou University Hospital (AJIRB-MED-SUR-19-261). Informed consents were obtained from all participants.

### Diagnosis of the TMD and determination of the number of myofascial trigger points (TrPs)

TMD was diagnosed according to the Diagnostic Criteria for/TMD (DC/TMD) criteria [15]. Clinical parameters, such as the extent of pain free opening and maximum unassisted opening, as well as the duration of TMD symptoms, including TMJ noise, difficulties in opening and/or closing the mouth, and pain in the jaw, temple and preauricular areas were evaluated. A visual analogue scale (VAS) and Global Chronic Pain Scale (GCPS) based on the DC/TMD axis II were applied to assess the subjective severity of the orofacial pain. Myofascial TrPs were bilaterally evaluated in the two masticatory muscles including the temporalis and masseter muscles. TrPs were determined in accordance with the criteria suggested by Simon and Travell [16]. The parameters which were assessed by one TMD specialist (JHK) such as extent of pain free opening and maximum unassisted opening and number of active and latent TrPs in masticatory muscles were regarded as objective outcome variables. One examiner (JHK) repeated same procedures for determining extent of pain free opening and maximum unassisted opening and number of active and latent TrPs in masticatory muscles in 20 participants after 2 weeks and the data were compared using intraclass correlation coefficient (ICC) to assess the intra-examiner reliability. The ICC was 0.906 with statistical significance. The parameters which were determined by self-administered questionnaires such as VAS and GCPS scores were considered as subjective outcome variables.

### Psychological evaluation

To assess the confounding factors for TMD treatment, the Symptom Checklist-90-Revised (SCL-90-R) [17] was applied. The SCL-90-R is a tool designed to assess psychological conditions. The instrument consists of 90 questions and includes nine symptomatic dimensions, such as somatization (SOM), obsessive–compulsive (O–C), interpersonal sensitivity (I–S), depression (DEP), anxiety (ANX), hostility (HOS), phobic anxiety (PHOB), paranoid ideation (PAR), and psychoticism (PSY) and three global functioning indices, such as the global severity index, positive symptom distress index, and the positive symptom total. The T scores for the symptomatic dimensions were utilized.

### Assessment of the participants’ roles in decision-making

The Control Preferences Scale (CPS) was used to evaluate the roles that participants preferred to play or had perceived themselves as playing in actual decision-making during the six months of TMD management [18]. The CPS consists of five items that determine different roles in decision-making. Participants ranked ordered possible approaches of decision making from 1 (most active) to 5 (most passive). The perceived roles which participants had perceived during the decision-making were categorized in to 3 groups: active-A (CPS 1–2), collaborative-A (CPS 3), and passive-A (CPS 4–5) [19]. The 5 different roles which participants preferred during the decision-making were also re-grouped into 3 groups that active-P (CPS 1–2), collaborative-P (CPS 3), and passive-P (CPS 4–5) [19]. The evaluation of perceived and preferred role in decision-making were conducted at T1.

### Measurement of health literacy

The Newest Vital Sign (NVS) was used to assess the participants’ levels of health literacy [20]. The NVS health literacy test is based on a nutrition label from an ice cream container, and the overall score ranges from 0 to 6. Participates were categorized as having limited (NVS score of 0–2), potentially limited (NVS score of 3), and appropriate (NVS score of 4–6). The assessment of level of health literacy was performed at T1.

### Management of TMD

The participants who performed stabilization splint therapy and physical therapy for six months to manage the orofacial pain and parafunctional habits for six months were included in the study. The participants who showed limited range of jaw movement owing to disc displacement, sustained pain around masticatory muscles or preauricular area despite of 2 weeks of treatments including medication and habit control, and pathologic bony changes on TMJ condyles were prescribed stabilization splint therapy. All participants were instructed to wear their stabilization splints every night for at least seven hours per day during the treatment period. All participants were instructed to control for TMD-related contributing factors including excessive parafunctional habits, masticatory muscle tension, and conduct routine physical therapy such as 6 × 6 exercises [21] and moist hot pack application. The stabilization splint which covered all of the maxillary teeth was fabricated in acrylic resin. Even uniform contact of the stabilization splint with the lower functional cusps was provided at premolar and molar teeth sites. Continuous wearing of stabilization splint more than seven hours per day was regarded as valid wearing and considered as compliant with stabilization splint therapy. All participants visited the clinic monthly for routine check-ups and confirmation of compliance.

### Statistical analysis

Power analysis indicated that a total sample size of 131 participants in the F test with 3 groups and 2 times repetitions would provide a statistical power of 92.1% at a 0.05 significance level with an effect size of 0.25. Based on the Shapiro–Wilk normality test, the data were found to be normally distributed; therefore, parametric tests were applied.

To compare the differences of demographic characteristics and parameters related with TMD of the participants including age, sex distribution, body mass index (BMI), education level, and classification of TMD based on DC/TMD criteria, duration of TMD, amount of pain free opening and maximum unassisted opening, number of active and latent TrPs in masticatory muscles, GCPS, and VAS among three groups, Acitve A, Collaborate-A, and Passive-A, one-way analysis of variance (ANOVA) and chi-square test were applied for continuous and categorical variables, respectively. Chi-square test was used to determine statistical differences of levels of preferred and perceived level of decision-making role and educational level accordance with the levels of health literacy. To estimate the responses of differences of the subjective and objective TMD treatment outcomes in accordance with the perceived role in decision-making, two-way repeated measure ANOVA was applied.

## Results

### Demographic characteristics and features of the TMDs of the participants

There were no significant differences in BMI (*P* = 0.334), sex distribution (*P* = 0.159), duration of TMD (*P* = 0.395), distribution of DC/TMD diagnosis, amount of of pain free opening (*P* = 0.941) and maximum unassisted opening (*P* = 0.630), number of active (*P* = 0.060) and latent TrPs (*P* = 0.066) in the masseter and temporalis muscles, levels of GCPS (*P* = 0.180) and VAS (*P* = 0.092) among the groups in accordance with the levels of perceived roles in decision-making. Otherwise, significant differences of age (*P* = 0.002) and educational levels (*P* = 0.024) were detected among groups in accordance with the perceived roles in decision-making (Table 1). No significant differences of SCL-90-R scores were detected among three groups (Additional file 1: Table S1).

**Table 1:** Demographic characteristics and features of TMD at baseline (T0)

	Active-A (n = 64)	Collaborate-A (n = 14)	Passive-A (n = 53)	*P* value
Age (years)	33.4 ± 13.1	38.6 ± 14.4	42.8 ± 15.1	0.002*
Sex (male/female) ^†^	6/58	0/14	9/44	0.159
BMI	21.1 ± 2.9	21.2 ± 3.5	21.9 ± 2.8	0.334
Education level (elementary/middle school/high school/university)	0/6/17/41	0/0/3/11	3/0/22/28	0.023*
TMD onset (months)	21.2 ± 31.3	38.2 ± 67.3	26.6 ± 47.5	0.395
*Classification based on DC/TMD criteria*				
Myalgia/MFP/Arthralgia /Myalgia + Arthralgia /Myofascial pain + Arthraltia^†^	16/6/11/13/10/8	4/2/2/3/2/1	13/4/3/12/9/12	0.762
Headache attributed to TMD (yes/no) ^†^	49/9	9/3	45/8	0.687
Normal disc /DD with reduction /DD with reduction with intermittent locking /DD w/o reduction with limited opening /DD w/o reduction without limited opening /subluxation^†‡^	28/36/22/16/26/0	4/9/1/6/7/1	29/27/9/12/22/7	0.054
Amount of pain free opening (mm)	42.6 ± 8.1	42.4 ± 8.9	42.0 ± 8.5	0.941
Amount of maximum unassisted opening (mm)	45.2 ± 6.3	45.1 ± 7.3	44.1 ± 6.7	0.630
Number of active TrPs in masticatory muscles	0.75 ± 1.32	1.30 ± 1.83	0.43 ± 0.97	0.060
Number of latent TrPs in masticatory muscles	0.69 ± 1.08	0.79 ± 0.97	0.66 ± 0.62	0.066
GCPS^†^	3 (1–3)	2 (1–3.25)	1 (1–3)	0.180
VAS	4.70 ± 2.31	4.64 ± 1.98	3.68 ± 2.68	0.092

Descriptive values are shown as mean ± SD or median (25–75th percentile)^†^

Data obtained from one-way ANOVA

*BMI* Body mass index; *TMD* Temporomandibular disorders; *GCPS* Graded chronic pain scale, *TrP* Trigger points; *VAS* Visual analog scale

^†^Data obtained from chi square test

^‡^The diagnosis of intra-articular TMD was conducted separately in both side of the TMJs

*P < 0.05 by one-way ANOVA and Chi square test

### Levels of health literacy and perceived role in decision-making

The distributions of perceived (*P* = 0.002) decision-making role showed significant differences accordance with the levels of health literacy. On the other hand, the preferred role in decision-making did not show statistical significance accordance with the degree of health literacy (*P* = 0.053). The level of institutionalized education did not show direct relationships with degrees of health literacy (*P* = 0.151) (Table 2), but it seemed to have strong association with perceived decision-making role (*P* = 0.023) (Table 1).

**Table 2:** Differences of preferred and perceived participation in decision-making along with the level of health literacy

	Limited (n = 28)	Potentially limited (n = 43)	Appropriate (n = 60)	*P* value
Preferred decision-making role (Acitive-A/Collaborate-A/Passive-A)	10/5/13	21/2/20	38/6/16	0.053
Perceived decision-making role (Active-P/Collaborate-P/Passive-P)	7/3/18	17/6/20	40/5/15	0.002*
Education level (elementary/middle school/high school/university)	1/1/9/17	2/0/18/23	0/5/15/39/60	0.151

Data obtained from chi square test

*P < 0.05 by Chi square test

### The influences of role of perceived decision-making on the treatment outcomes of TMD

The results from two-way repeated measure ANOVA demonstrated that there existed statistically significant relationships between time and groups in accordance with the patients’ perceived role in decision-making in VAS. On the other hand, the amount of pain free and maximum unassisted mouth opening and number of active and latent TrPs in the temporalis and masseter muscles did not show significant relationships. The distribution of GCPS showed significant differences among the groups but no differences were found among the groups in accordance with the extent of perceived decision-making (Table 3). Hence, the improvement of objective signs which assessed by TMD specialist such as amount of pain free and maximum unassisted maximum mouth opening and number of active and latent TrPs did not show significant differences but the subjective outcome variables which determined by self-administered questionnaire, including VAS and GCPS showed significant differences along with the level of perceived decision-making role.

**Table 3:** Differences of six months of treatment outcome of TMDs accordance with the level of perceived role of decision-making

		Baseline	6 months after treatment	*P* value		
				Time	Group	Group*Time interaction
Amount of pain free opening (mm)	Active-A	42.6 ± 8.1	47.6 ± 5.9	< 0.001**	0.185	0.692
	Collaborate-A	42.4 ± 8.9	47.1 ± 5.8			
	Passive-A	42.0 ± 8.5	47.1 ± 6.5			
Amount of maximum unassisted opening (mm)	Active-A	45.3 ± 6.3	47.7 ± 6.0	0.006*	0.183	0.658
	Collaborate-A	45.1 ± 7.3	47.3 ± 5.5			
	Passive-A	44.1 ± 6.7	47.6 ± 5.8			
VAS for TMD	Active-A	4.70 ± 2.61	1.17 ± 1.4	< 0.001**	0.311	< 0.001**
	Collaborate-A	4.64 ± 1.98	2.62 ± 1.98			
	Passive-A	3.68 ± 2.68	4.00 ± 2.39			
Number of active TrPs in masticatory muscles	Active-A	0.75 ± 1.32	0.20 ± 0.69	< 0.001**	0.339	0.771
	Collaborate-A	1.03 ± 1.83	0.43 ± 1.16			
	Passive-A	0.43 ± 0.97	0.04 ± 0.19			
Number of latent TrPs in masticatory muscles	Active-A	0.69 ± 1.08	0.44 ± 0.90	0.196	0.280	0.098
	Collaborate-A	0.79 ± 0.97	0.14 ± 0.53			
	Passive-A	0.26 ± 0.62	0.26 ± 0.76			
GCPS^†^	Active-A	3 (1–3)	1 (0–1)	0.006*	0.235	–
	Collaborate-A	2 (1–3.25)	1 (0.5–2)			
	Passive-A	1 (1–3)	2 (1–3)			

Descriptive values are shown as mean ± SD or median (25^th^ – 75^th^ percentile)^†^

Data obtained from two-way repeated measure ANOVA

*TMD* Temporomandibular disorders; *GCPS* Graded chronic pain scale; *TrP* Trigger point; *VAS* Visual analog scale

^†^Data obtained from Chi-square test

*P < 0.05, **P < 0.001 by two-way repeated measure ANOVA and Chi-square test

## Discussion

Considering the complex etiology and pathophysiology of the TMDs, educating the patients to promote their understanding of the properties of their conditions and helping them to make proper medical decisions are critical to the successful management of TMD. Shared decision-making procedures through which the physicians and informed patients make a shared medical decision regarding the values and preferences of the patients [2] can improve both clinicians’ and patients’ satisfaction leading to better treatment outcomes [2–4]. Moreover, the active participation of patients in decision-making is important to limit the potential of misunderstandings of patients’ preferences and decision conflicts between clinicians and patients [6]. However, patients’ levels of health literacy would have an impact on the patients’ active participation in treatment procedures and decision-making [6, 8–11, 13, 14]. To the best of the knowledge, sparse studies ever have investigated the roles of shared decision-making and levels of health literacy with respect to treatment outcomes and the satisfaction of dentists and patients in TMD management. Therefore, the purpose of the present study was to determine the impacts of TMD patients’ preferred and perceived roles in shared decision-making and patients’ degrees of health literacy on the outcomes of a TMD treatment.

The main finding of the present study was that the extent of subjective symptomatic improvement after six months of treatment presented significant difference in accordance with the level of perceived role in decision-making, despite a lack of significant differences regarding the extent of improvement of the objective signs including amount of pain free and maximum unassisted mouth opening and number of TrPs in masticatory muscles. The VAS and GCPS scores showed prominent improvement in participants with active and collaborative perceived roles in decision-making, while those with passive roles in decision-making showed symptomatic worsening. Previous studies showed similar results in which bidirectional shared decision-making could improve surgical outcomes as well as increase satisfaction in patients with strabismus or prostate cancer [22, 23]. Those studies suggested that when physicians presented a wide range of information and encouraged patients to be actively involved in the decision-making process, the patients’ effective decision-making would be conducted and, consequently, the patients’ overall treatment satisfaction would be increased. These findings could be applied to TMD treatment, also. Regarding the complexity of the condition and long treatment period, helping the patients to understand their health conditions and facilitating their active participation in treatment procedures through shared decision-making could improve patients’ treatment satisfaction and improve their TMD management outcomes.

The aforementioned results showed an interesting point that even though the participants with limited health literacy played passive perceived roles in decision-making, they preferred active participation in their treatment. Previous studies already have reported that a limited understanding of medical knowledge, owing to a patient’s low level of health literacy, could act as a barrier for active participation in a treatment [8–10, 12, 13]. Therefore, several attempts for developing and offering decision aids for patients with low levels of health literacy by allowing them to be more informed and more involved in shared decision-making have been tried [24–26]. Those studies showed that such decision aids significantly encouraged patients’ active participation in decision-making and resulted in improvements to patients’ satisfaction. Hence, the screening for background levels of health literacy and considering for offering proper types of decision aids such as videos or leaflets to promote an understanding of the disease, before TMD treatment are necessary for the successful TMD management.

To the best of the knowledge, the present study is the first attempt to reveal the impacts of shared decision-making in treatment outcomes and satisfaction of patients in TMD treatments. However, there are several limitations of the study. First of all, due to the characteristics of the TMD, few male participants were included, so limited information was provided. Second, all participants were recruited from a tertiary medical center, which might have resulted in the collection of distorted information about the shared decision-making and health literacy as compared information collected from ordinary individuals with TMDs. Thirdly, small sample sizes would inevitably lessen the validity of the results. Finally, even though the cultural background could critically influence on patients’ attitude and active participation during treatment, this study could not give meaningful information about that because only Koreans were included in the study. Therefore, future community-based studies with large sample sizes which include a sufficient number of male participants and participants with diverse ethnicity are needed. Moreover, attempts to develop appropriate forms of decision aid to help patients with low levels of health by allowing them to be more informed and more involved in shared decision-making should be tried.

## Conclusion

Hence, active participation in decision-making improves the satisfaction of TMD patients, whereas limited health literacy constitutes a barrier to effective patient engagement and shared-decision making in TMD management. Furthermore, further investigation of ways to inform patients with limited health literacy and encourage their active participation in shared decision-making in TMD management is warranted.

## Supplementary Information

**Additional file 1: Table S1.** The differences of psychological evaluation in accordance with level of perceived decision-making.

## Data Availability

The datasets used and/or analyzed during the current study are available from the corresponding author on reasonable request.

## Abbreviations

ANOVA: Analysis of variance
BMI: Body mass index
CPS: Control preferences scale
DC/TMD: Diagnostic criteria for temporomandibular disorders
GCPS: Global chronic pain scale
NVS: Newest vital sign
SCL-90-R: Symptom checklist-90-revised
TMD: Temporomandibular disorders
TrPs: Trigger points
VAS: Visual analogue scale
