# Supplementary material for: Influences of decision preferences and health literacy on temporomandibular disorder treatment outcome
Source: BMC Oral Health. 2022 Sep 5;22:385. doi: 10.1186/s12903-022-02420-x (PMC9446804; doi:10.1186/s12903-022-02420-x)
Supplement: Supplementary file 1 — Additional file 1: Table S1. The differences of psychological evaluation in accordance with level of perceived decision-making. [file 12903_2022_2420_MOESM1_ESM.docx]

**Supplementary Table 1.** The differences of psychological evaluation in accordance with level of perceived decision-making

|  | Active-A  (n = 64) | Collaborate-A  (n = 14) | Passive-A  (n = 53) | *P* value |
| --- | --- | --- | --- | --- |
| SOM | 46.9 ± 7.5 | 49.9 ± 12.8 | 47.3 ± 6.2 | 0.054 |
| O-C | 40.8 ± 7.6 | 42.4 ± 8.9 | 40.0 ± 7.3 | 0.567 |
| I-S | 41.3 ± 7.8 | 42.4 ± 10.0 | 41.1 ± 6.1 | 0.848 |
| DEP | 40.9 ± 7.4 | 42.1 ± 7.5 | 40.5 ± 5.7 | 0.718 |
| ANX | 43.2 ± 7.0 | 44.7 ± 6.5 | 42.4 ± 4.9 | 0.421 |
| HOS | 43.1 ± 4.8 | 44.2 ± 7.8 | 42.8 ± 4.2 | 0.653 |
| PHOB | 44.7 ± 3.7 | 47.1 ± 10.0 | 44.3 ± 3.0 | 0.119 |
| PAR | 40.5 ± 5.0 | 41.6 ± 6.9 | 39.9 ± 4.8 | 0.565 |
| PSY | 42.2 ± 5.6 | 44.9 ± 11.1 | 42.0 ± 5.1 | 0.281 |
| GSI | 41.5 ± 7.1 | 43.4 ± 9.9 | 40.0 ± 7.2 | 0.280 |
| PSDI | 44.2 ± 7.6 | 43.2 ± 8.1 | 43.4 ± 6.0 | 0.807 |
| PST | 39.1 ± 9.6 | 42.4 ± 10.6 | 38.8 ± 8.6 | 0.412 |

SOM, somatization; O-C, obsessive-compulsive; I-S, interpersonal sensitivity; DEP, depression; ANX, anxiety; HOS, hostility; PHOB, phobic anxiety; PAR, paranoid ideation; PSY, psychoticism; GSI, global severity index; PSDI, positive symptom distress index; PST, positive symptom total

Descriptive values are shown as mean ± SD.

Data obtained from one-way ANOVA.

*^*^P <* 0.05 by one-way ANOVA.
